# Exceptional improvement in chronic stroke through Guided Self-rehabilitation Contract: a case report study

**DOI:** 10.3389/fresc.2024.1385483

**Published:** 2024-09-18

**Authors:** Caroline Gault-Colas, Maud Pradines, Marjolaine Baude, Jean-Michel Gracies

**Affiliations:** ^1^ AP-HP, Service de Rééducation Neurolocomotrice, Unité de Neurorééducation, Hôpitaux Universitaires Henri Mondor, Créteil, France; ^2^ EA 7377 BIOTN, Laboratoire Analyse et Restauration du Mouvement, Université Paris Est Créteil (UPEC), Créteil, France

**Keywords:** stroke, chronic, hemiparesis, self-rehabilitation, guided self-rehabilitation contracts, spastic paresis, upper limb, case report

## Abstract

A 44-year-old woman suffered a carotid dissection causing a deep and superficial right middle cerebral artery stroke in October 2013, despite undergoing thrombolysis and thrombectomy. Sixteen months later, massive left upper extremity impairment persisted. She then agreed to embark upon a guided self-rehabilitation contract (GSC). This GSC is a moral contract where the physician or therapist identifies specific muscles, particularly hypo-extensible and disabling that act as antagonists to functional activities. The physician or therapist then teaches and prescribes quantified daily high-load self-stretch postures for these muscles, alternating with repeated maximal amplitude movement exercises against their resistance. In turn, the patient commits to practicing the prescribed program and to delivering a diary of the stretch postures and alternating movement exercises performed each day. Over 4 years of GSC, the patient practiced upon prescription against a total of seven upper limb antagonists to common functional movements: shoulder extensors, shoulder internal rotators, elbow flexors, elbow pronators, wrist and finger flexors, and interossei muscles. She manually filled up her diary 99% of the days. Each day, she practiced an average of 20 min of high-load static self-stretch per muscle, alternating with about 50 maximal active efforts against the resistance of each targeted muscle's resistance. Overall, her mean static self-stretch time was 81 ± 2 (mean ± SEM) min/day, and her mean number of active maximal efforts was 285 ± 78/day, for a total daily self-rehabilitation time of over 2 h a day. Five years after her stroke, she had recovered all left upper extremity use in daily activities and resumed her previous job as a nurse's aide. She now spontaneously uses her left hand in most tasks. Functional MRI (March 2020) demonstrated bilateral primary motor and motor supplementary area activation upon left-hand exercise. Prolonged static self-stretch increased muscle extensibility (muscle plasticity) while maximal amplitude, alternating movement training reduced co-contraction in these muscles (neural plasticity). The Modified Frenchay Scale assessment was video-recorded by the clinician at each visit, allowing qualitative and quantitative evaluation of the functional capacities. The two videos of the first and last clinic visits have been uploaded and are available.

## Introduction

The syndrome of spastic paresis occurs following a lesion involving the central motor pathways that process the execution of voluntary motor command (1, 2). The main cause is stroke, with approximately 70% of stroke survivors experiencing an initial motor deficit, with walking recovery possible in most cases (3). In contrast, only 10%–20% of stroke patients recover “normal” upper limb function, i.e., the ability to use the paretic hand in daily activities during chronic stages (>6 months) (3–9). This represents a major challenge for the rehabilitation community. Thus, there is an important need for rehabilitative approaches capable of enhancing upper limb motor recovery for stroke patients (10).

Conventional rehabilitation after stroke has included sensorimotor training, strengthening, or task-oriented exercises (11). Other techniques such as constraint-induced movement therapy (CIMT) or mirror therapy have been promoted (12, 13). More recently, technological advances have allowed upper limb exoskeletons, robot-assisted therapies, and virtual reality to also emerge (14, 15). Although research in these areas has produced preliminary results, the high cost of these devices limits their accessibility globally.

Meanwhile, today, there is a better appreciation of the various determinants of neuronal plasticity. For patients with spastic paresis, it is now understood that the *dose* of any rehabilitation intervention may be a more powerful stimulus to improving motor function than the exact rehabilitation technique (16).

However, in chronic stages, our current healthcare systems are unable to provide sufficient time for physical therapy through prescriptions (17). After discharge from rehabilitation units, the amount of delivered therapy typically decreases over time. Under these conditions, recovery of motor function is classically limited at the chronic phase, often improperly interpreted as having reached a form of ineluctable “plateau,” as if no further progress were then possible (6). Yet, this interpretation reflects confusion between two types of plasticity, lesion-induced plasticity, which indeed is short-lived, lasting a few months only (18), and behavior-induced plasticity, which remains available all life long (19). Therefore, recent recommendations for stroke rehabilitation advocate for intensive, long-lasting, challenging, repetitive, and motivating training (20, 21), with high doses needed to maximize neural recovery (16).

The strategy of *guided self-rehabilitation contract* [GSC, (22)] aims to meet the criteria of intensity through two principles, namely, *psychological* and *technical*. GSC is a diary-based rehabilitation system targeting antagonistic muscles (22). It relies on a moral contract between patient and therapist. The GSC therapist (e.g., doctor or physical or occupational therapist) prescribes and teaches a daily program of self-stretching postures and maximal alternating movement exercises targeted to the muscles found to be the most disabling at the five-step assessment (FSA) (see below) and provides the patient with the GSC manual (22). During each follow-up encounter, the GSC clinician corrects and adjusts the program according to progression.

The technical principle of GSC requires, from the clinician, a clear understanding of the pathophysiological processes involved to identify the muscle and command disorders that need treatment in chronic spastic paresis in particular. Pathophysiological understanding of the syndrome of spastic paresis revolves around the characterization of two disorders: spastic myopathy (1, 2, 23, 24), which appears early in the acute phase (loss of extensibility of muscles immobilized in a short position) and then only worsens, and the motor command disorder, due to later emerging plastic neural mechanisms causing motor neuronal overactivity that mostly involves the shortened antagonists (1, 2, 25, 26). One notable form of motor neuronal overactivity is spastic co-contraction, a misdirection of the supraspinal descending drive that fires motor neurons that are antagonists to the desired movement (1, 2, 25–27).

In chronic stages, spastic overactivity is then superimposed on muscle changes, with reciprocal potentiation between the two (1, 2, 22–26). These muscular and neural phenomena coalesce to produce agonist–antagonist imbalance around joints, with spastic co-contraction, in particular, directly impeding and sometimes reversing the desired voluntary movements (25–27). In subacute and chronic stages after a stroke, active movement becomes primarily hindered by these antagonist resistances, rather than by agonist paresis (1, 2, 25–27). In the upper limb, typical antagonists to daily functional movements are shoulder extensors, shoulder internal rotators, elbow flexors, pronators, wrist flexors, finger flexors, and interosseous muscles (28). There is a need to *estimate* spastic myopathy and muscle overactivity (spastic co-contraction) in such antagonist muscles, which can be achieved through the five-step assessment [see below, (23, 29, 30)].

From a therapeutic point of view, to tackle muscle shortening and excessive co-contractions, there are two key physical techniques. Daily high-load self-stretch postures (for muscles specifically selected by the prescribing clinician) stimulate muscle plasticity, and alternating with repeated maximal amplitude movement exercises against these antagonists stimulates neural plasticity (22, 31–33). The stretching program involves prolonged static self-stretching postures, >15 min/day/muscle at high load—just below pain threshold—for selected specific antagonists. The training program consists of a series of alternating efforts of maximum amplitude against each targeted antagonist, for short periods of time (10–30 s/series, adapted to fatigability) to decrease antagonist co-contractions over time (33, 34).

The psychological principle of GSC uses a strategy to enhance and maintain the motivation level of the patient over the long term, exploiting a tool of quantitative self-monitoring, the quantified *diary* (35–37). The patient performs the prescribed program on a daily basis and keeps a log quantifying the work actually accomplished every day, specifying the duration of self-stretching postures and the number of efforts or movements performed with each series of rapid alternating movements (when no work is actually done on a given day, the patient must write *zero* in the relevant boxes). The diary may be better in paper form (notebook, binder, etc.) or may be computerized. It must be turned to the GSC therapist at each encounter and enables the therapist to assess the work actually performed and the compliance with the program. The GSC therapist then provides *frequent* enough quantified assessments (i.e., from once a week to once a month, depending on the patient’s understanding of the program and compliance) to the patient to demonstrate clinical progress, correct the exercises performed by the patient, and adjust the program over time according to clinical evolution. Once such careful, frequent enough, quantitative estimations (23, 29, 30) are regularly generated, we observe that the GSC ends up motivating patients to apply intensive daily practice at home, increasing the amount of physical work compared to conventional systems (17). In a recent controlled trial, a 1-year GSC program targeting plantar flexors in patients with chronic hemiparesis did produce structural changes in these muscles and improved walking speed as compared with conventional rehabilitation. In this study, the patient devoted at least twice as much time to home rehabilitation than with conventional community-based physical therapy (17).

Here, from massive hemiparesis after a large ischemic stroke, we describe near-perfect upper limb motor recovery in chronic stages through a 4-year GSC (22).

## Case presentation

### Patient information: initial assessment and prescription

**Figure 1:**
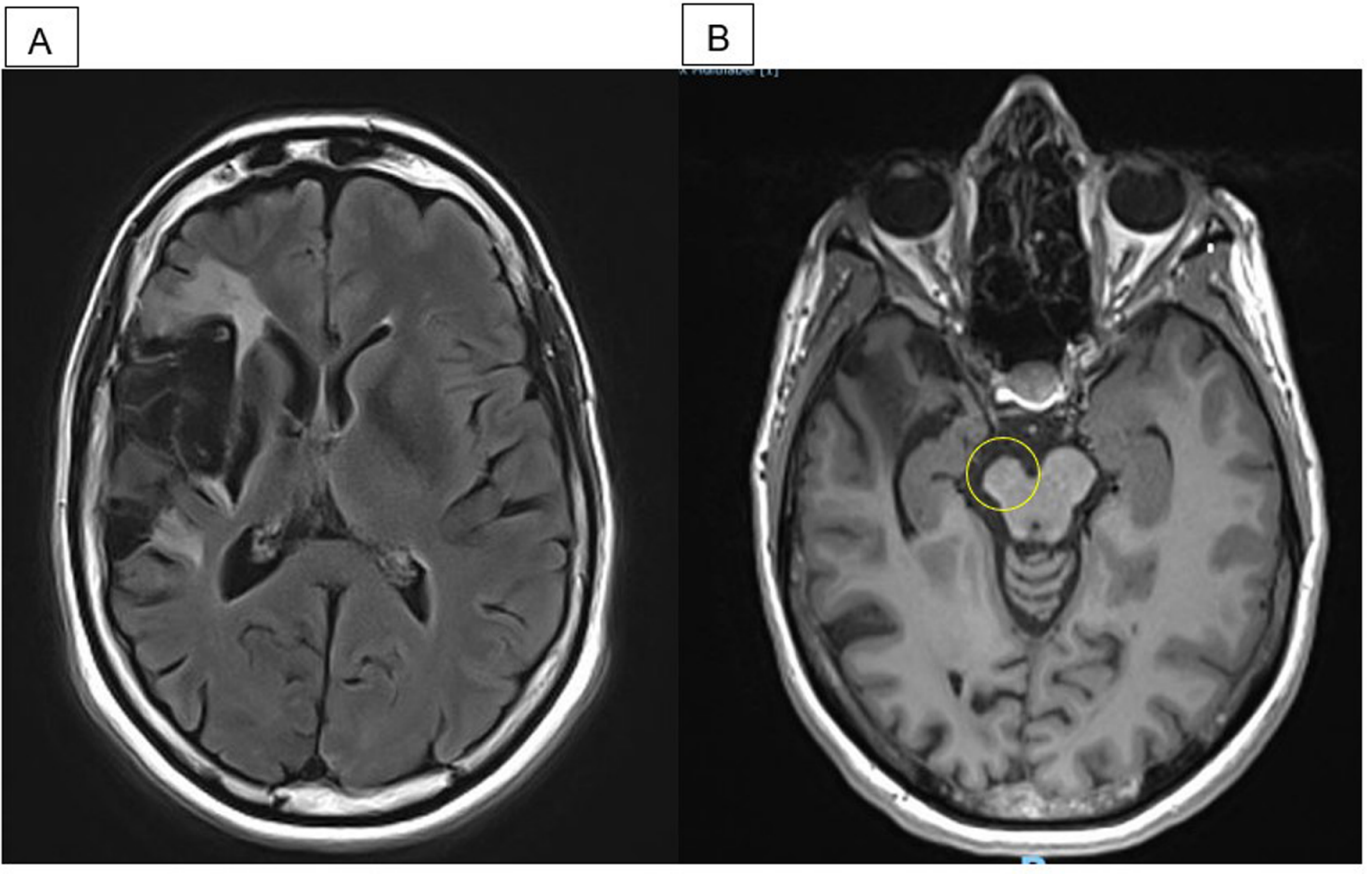
MRI performed in April 2019, 5.5 years poststroke and 4 months after the end of guided self-rehabilitation contract (GSC). **(A)** Axial flair: right middle cerebral artery infarct. **(B)** Axial T1: right midbrain atrophy.

A 44-year-old woman with no prior medical history suffered a carotid dissection leading to a large superficial and deep right middle cerebral stroke in October 2013 (Figures 1A,B), despite undergoing thrombolysis and thrombectomy. Sixteen months poststroke, severe left hemiparesis persisted. The rehabilitation she had received up until then had been carried out during conventional inpatient hospitalization in another center for 4.5 months (physical therapy 2 h/day and occupational therapy 2 h/day). Following this, she continued with outpatient rehabilitation for 3 days/week for 6 weeks (physical therapy 2 h/day and occupational therapy 2 h/day, i.e., 12 h/week). During her hospitalization, she also benefited from one botulinum toxin injection into elbow and finger flexors. At the time of the first clinic visit to our GSC center, 16 months poststroke, she had been receiving typical low-intensity community-based physical therapy, 45 min 3 times a week since 6 months poststroke.

At this initial assessment in our GSC center, the Modified Frenchay Scale (MFS), which involves video recording of 10 daily activities—6 bimanual and 4 manual with the paretic hand (38)—yielded a score of 3.5/10, indicating poor functional status (see Supplementary Video 1 and Supplementary Appendix—Explanations of Videos and MFS). Technical steps of the five-step assessment (FSA) were then carried out in the paretic upper limb to estimate passive (X_V1_) and active (X_A_) resistances in each antagonist [see Supplementary Appendix—Finger Flexor Assessment; (23, 29, 30)]. Four main antagonistic muscles limiting function were first identified and targeted through the initial prescription: shoulder extensors and internal rotators and wrist and finger flexors (Supplementary Table 1).

### GSC frame and techniques

**Figure 2:**
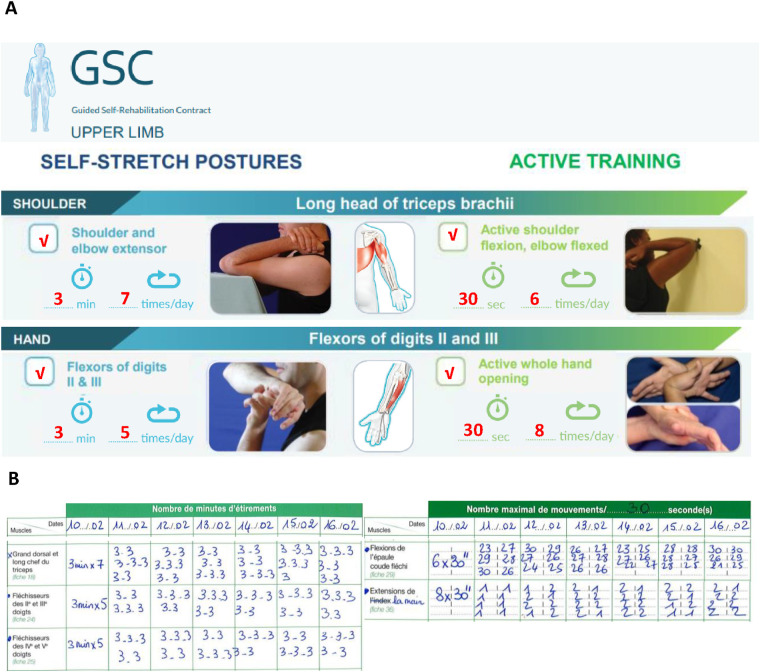
Guided self-rehabilitation contract (GSC) with excerpts from the prescription and from the patient's diary. **(A)** GSC prescription showing, on the left, the antagonist muscles to be stretched (daily time and frequency) and, on the right, the series of active exercises to be performed against each of these antagonist muscles; here examples of the long head of triceps and flexors of digits II and III (daily time and frequency) prescription. Working against the resistance of each selected antagonist muscle implies alternating between stretching time and a training series, with the patient starting and finishing with a stretching time. **(B)** Photo of patient's diary: on the left, the patient recorded the number of minutes static self-stretch was performed daily against the selected antagonist, and on the right, the number of active maximal efforts performed in each series against the resistance of the selected antagonist.

A GSC was then agreed upon with the patient, involving a first prescription of homework of about 1 h/day, according to the targeted objectives/antagonists. The exercise techniques were explained and demonstrated to her during the first clinic visit, and she was given the GSC manual (22) together with a paper GSC prescription (Figure 2). The patient was asked to keep a diary quantifying the homework done each day, which was to be reported at each clinic visit. Figures 2A,B show the excerpts of the prescription and of the diary later turned in by the patient.

### Follow-up and detailed data on the 4-year GSC program

Psychologically, the patient demonstrated high levels of intrinsic motivation right from the start of the GSC and unfailing determination throughout the follow-up, well supported by her husband. This motivation was reinforced by the regular medical monitoring with the GSC clinician.

In addition, within 3 months of starting the GSC, the patient and her husband participated in two small-group GSC educational workshops (four patients, duration 2 h) led by a GSC-certified physical therapist. These workshops allowed the patients to better understand the pathophysiological basis and the principles of application of the GSC and also taught them to practically perform the stretch and alternating movement techniques in their program, adapted to their capacities and home environment (39).

#### Medical visits

The psychological and technical importance of the long and detailed clinic visits cannot be overemphasized. Indeed, the identification of a small increase in functional capacities through the Modified Frenchay Score (±0.10; 38) and the relevance of changes in the clinical evolution for each quantified step (X_V1_, X_A_, and other steps of the FSA, ±3°; 17) in each muscle, detecting a progression of a few degrees, are of fundamental importance, with regard both to technical and psychological aspects of the GSC frame. All quantified clinical assessments during the 4-year follow-up were carried out by a rehabilitation physician (CGC) trained in the five-step assessment and in the GSC method, amounting to 27 long-duration medical clinic visits (2 h per visit on average) with an average visit-to-visit interval of 52 ± 12 days.

As stated above, these long visits systematically included the Modified Frenchay Scale (MFS) (38) and a technical assessment of antagonistic muscles (23, 29, 30). They also included an analysis of the diary and correction of the exercise techniques and ended up with adjustments to the GSC program according to the targeted objectives. The patient was accompanied on each occasion by her spouse and twice by a close relative as well.

#### Evolution of the prescription

**Figure 3:**
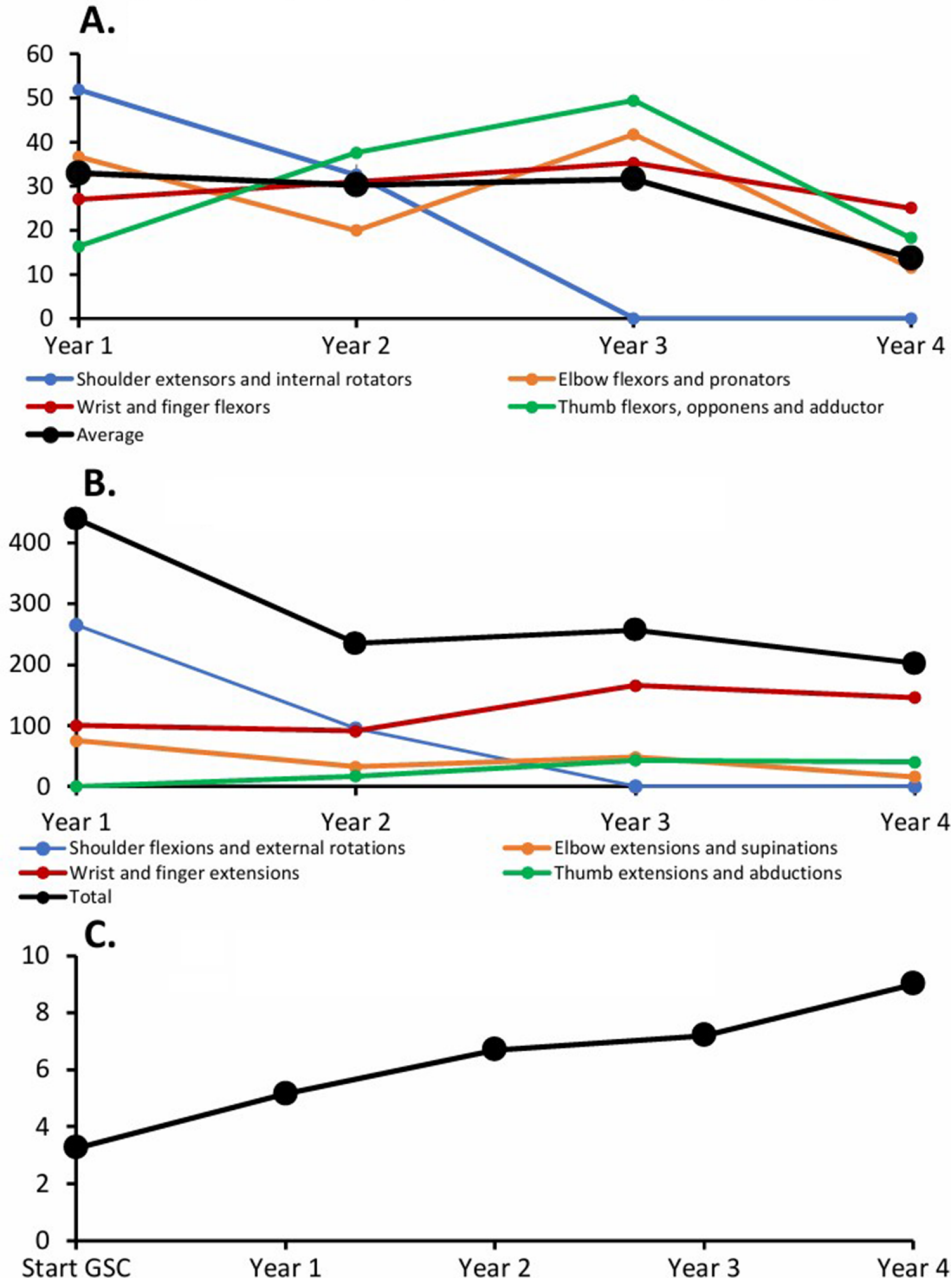
Individual data—exemplary work. **(A)** Static self-stretch time per muscle (min/day). **(B)** Number of movements/day. **(C)** Modified Frenchay Scale (MFS).

The GSC was progressively intensified in terms of duration and frequency of the prescribed exercises, over the 4-year program. Over time, the complete GSC training program ended up focusing on a total of seven of her most shortened and overactive antagonistic muscles: shoulder extensors and internal rotators, pronators, elbow flexors, wrist and finger extensors, and interossei. Exercises gradually evolved in a proximal–distal fashion, from shoulder muscles to pronators and wrist and finger flexors. Among the prescribed antagonist muscles, shoulder *extensors* and *internal rotators* represented the main part of the GSC self-rehabilitation program (60%) over the first year, reaching an amount of 52 min per day of self-stretch of these muscles and more than 265 active movements per day against those ones. During the second part of the GSC follow-up, pronators and wrist and finger flexor muscles gradually took over most of the guided self-rehabilitation efforts, with 30 min per day of self-stretch for finger flexors and more than 120 efforts per day of hand opening (Figures 3A,B).

#### GSC compliance

At each visit, the patient submitted a well-kept logbook, which the GSC clinician analyzed for program compliance and regularity. The patient performed her required work on 99% of the days, which was consistent with her strong motivation, with only 10 days without work over the entire 4-year period.

#### Adjunct treatments

During some clinic visits, the patient benefited from injections of botulinum toxin into shoulder extensors, shoulder internal rotators, elbow flexors, elbow pronators, and deep and superficial flexors of fingers and thumb to ease her stretching and active efforts to be made against the muscles. In addition, the patient supplemented her work program with 30 min per day of functional electrical stimulation on the finger extensors (self-applied using a rental device) during the first 2 years of GSC.

### Final assessment—quantitative results

Five years poststroke, functional recovery was complete, with an MFS score that had improved to 9/10 (see Supplementary Video 2 and Figure 3C).

All stretched antagonist muscles had regained adequate extensibility. After 2 years of work, the extensibility of shoulder extensors had increased from X_V1 _= 130° to X_V1 _= 180° (38% improvement), internal rotators from X_V1 _= 160° to 180° (+13%), wrist flexors from X_V1 _= 160° to 180° (+13%), and finger flexors from X_V1 _= 250° to 270° (+8%). Muscle overactivity of the antagonists had subsided, and active performance against each antagonist (X_A_) was roughly normalized. Specifically, active shoulder flexion had increased from X_A _= 90° to 180° (100% improvement), shoulder external rotation from X_A _= 100° to 180° (+80%), supination from X_A _= 90° to 180° (+100%), wrist extension from X_A _= 110° to 180° (+64%), and finger extension from X_A _= 0° to 270° (+270%; see Supplementary Table 1).

After 4 years of GSC, the patient had regained full spontaneous use of her upper limb. In terms of return to work, she actually had returned to work half-time in an adapted position a few weeks after the initial prescription. Four years into the GSC (close to 6 years after the stroke), she resumed her previous job as a full-time nurse's aide with no more activity restrictions.

Functional MRI (March 2020) demonstrated bilateral primary motor and motor supplementary area activation upon left-hand exercise.

## Discussion

In this case report, a daily customized program applied for 4 years through GSC, which targeted the most hindering antagonist muscles using high-load self-stretched postures and a series of alternating maximal efforts, resulted in exceptional functional improvement. The shortened and overactive antagonist muscles gradually increased their extensibility and reduced their spastic co-contractions, which led to gains in passive and active amplitudes in the upper limb slowly recovering physiologic values, as did the functional assessment measured using the MFS scale. The patient regained normal use of the upper limb “as if I’d never had a stroke” (see testimonial below) and resumed her full professional activity.

### Technical considerations pertaining to GSC: prolonged self-stretching and active, alternating effort training

#### Qualitative point of view

Technically, the GSC program aims to neutralize hindrance from specific antagonist muscles, targeting their various resistances through two types of rehabilitation techniques.

High-load prolonged static self-stretching (>15 min/muscle/day) prescribed to the patient addresses spastic myopathy. A sufficient time spent in the stretching posture at high load has been demonstrated to stimulate muscle plasticity in a stretching time-dependent manner, both in preclinical and clinical research to decrease passive resistance, treating spastic myopathy (17, 40, 41). Still, pessimistic conclusions may have been drawn regarding the efficacy of stretching in recent literature reviews (42). However, most of the reviewed studies were based on *short-term* experimentations (<6 months) that fell short of involving high-load *self-stretch* practice every day. We indeed believe that a properly guided self-stretch is likely to be more effective than a stretch performed by another person, even a therapist, because the intimate muscle sensations, particularly at high loads, are perceived *by the patient*, provided self-stretch has been well-instructed and well-guided (17, 22, 32). Furthermore, although conventional therapy comprises various stretching techniques, the derived stretching time has been shown to remain far from reaching the weekly amount of cumulative duration of stretch per muscle in GSC (17). In a rare long-term, controlled study of guided self-stretch, Pradines et al. (17) showed that a daily self-stretching program using GSC techniques with stretching postures combined with conventional rehabilitation over 1 year increased ultrasound measured plantar flexor fascicle length and clinical extensibility more than conventional therapy alone in patients with chronic hemiparesis.

Additionally, it has been shown that rehabilitation based on the practice of high numbers of repetitive tasks is a factor of functional improvements (43–48), promoting neural plasticity through synaptic sensitization (49), i.e.*,* the increase in the strength of synaptic connections (49–52). Series of rapid unassisted maximal efforts over a short time (10–30 s/series/day) have proven effective in gradually reducing co-contractions of the antagonist muscles (33, 34). Consequently, exercises within the GSC method include the repetition of large numbers of maximal efforts against the impeding antagonist.

Each prescribed series of exercises in the GSC plans involves passive self-stretching postures to be applied before and after each active series of alternating efforts.

#### Quantitative aspects: rehabilitation dose

In this case report, at the time of the initial consultation, 16 months poststroke, the patient had followed conventional rehabilitation sessions in private practice but complained that the work was not intense, wherein she had not felt the functional benefit. The dissatisfied patient, still hoping to make progress and pursue rehabilitation work, consulted with a physician trained in the five-step assessment and the GSC method. The practice of the self-rehabilitation program within the GSC frame began from the first clinic visit and proved able to restore markedly increased amounts of daily rehabilitation and to achieve functional progress at the chronic stage of stroke, breaking the two vicious circles *paresis-disuse-paresis* and *shortening-overactivity-shortening* (1, 2).

In the chronic phase after stroke, patients follow at best a “maintenance” therapy for 30 min from one to three times a week (i.e.*,* 11 mn/day; 17), partly because of the limited physical therapy care available in our healthcare systems. In addition, it has been shown that professionals themselves, in many cases, do not believe in their ability to promote further progress chronically after the lesion (53).

In our neurorehabilitation department, almost all chronic poststroke patients follow a GSC program with guided self-rehabilitation at home, from 20 min to more than 5 h per day (depending on motivation level, capacities, professional activity, or other constraints). Each of them progresses over the long term, at their own pace, as previously reported retrospectively (32). The patient reported here practiced over 2 h per day to reach such improvements. However, even without such involvement, therapists should not lose hope too quickly for patients with chronic hemiparesis and should develop individualized patient-centered rehabilitation interventions (16) favoring intensive rehabilitation to improve chronic function.

### Psychological approach in GSC

It has been shown that programs of exercises given by the therapist to be performed at home are appreciated by patients, not only for the structure they give to everyday life but also as they represent in themselves a source of motivation and hope, particularly when these programs are associated with ongoing professional support (54, 55).

From a psychological point of view, the patient needs to be sufficiently motivated to be able to maintain high levels of work intensity for such a long time. In this particular case, the patient demonstrated high levels of intrinsic motivation right from the start of the GSC (the fire-in-the-belly motivation is sometimes delayed to months after the GSC onset, depending on the situation and the patient’s personality). Here, the patient’s determination throughout the follow-up may have been strengthened by four major factors:

- *Regular and long clinic visits with the GSC clinician*. Indeed, consultations were frequent (<2 months of intervisit intervals), which lasted almost 2 h each and as stated systematically included as follows: quantified assessments (MFS within FSA) quantifying clinical evolution, including education through analysis of the diary, systematic review of exercise techniques, and adjustments of the GSC program according to the targeted objectives.
- *The support of the patient's spouse and family*. The patient's spouse and her close family were present during each consultation and encouraged her in everyday life regarding the application of the GSC program.
- *Participation in a few small-group workshops*. These sessions in small groups allow patients to meet each other for an hour or two, share their experiences, and practice together the prescribed intense exercises of their GSC program, which creates emulation between patients and a source of hope (39).
- *Completion of the diary.* Reporting to the patient a quantified monitoring of performance after stroke is itself therapeutic (56). Quantified *self*-monitoring through a diary adds the advantage of providing positive reinforcement to the patient (35–37). This tool also improves self-esteem and performance in other patient populations including the mentally disabled (57).

The specific framework of GSC between a therapist-*coach* and a patient-*actor responsibilizes* patients and encourages their long-term motivation. In fact, the GSC clinician should adopt an educational stance toward his patients, adjusting his/her behavior and tone according to the personality of each patient. Finding the words that the patient needs to hear to infuse motivation and hope is not an easy task, nor is it easy for the therapist to make patients aware of their own responsibility to make progress in the course of their therapy. However, patients gradually believe in themselves as a mirror image of what the clinician transmits to them (58). In our view, the *time* devoted by the clinician to the patient during clinical encounters and the faith in the patient are two key factors of success. A large-scale, multicenter, randomized controlled trial that aims to compare the effects of GSC to conventional community-based physical therapy on upper and lower limb motor function is underway (59).

## Conclusion

The GSC system makes it possible to combine daily intensity and long-term duration of rehabilitation work. This specific method is affordable at a global level for the greatest number of stroke patients. Referred to a GSC-trained therapist and provided with a specific manual and a customized prescription to be adjusted by the therapist, the patient can start a program right from the initial prescription. This case report offers promising prospects for the rehabilitation of chronic hemiparesis. If large-scale multicenter-controlled trials confirm the findings of this exceptional case, even partly, GSC may help reshape the way rehabilitation is conceived in chronic neurological disorders.

## Testimonial

According to the patient's own report during the final evaluation, 6 months after the end of the GSC, and in response to the questions asked by the doctor.

What would you say about the work you have done over the past 4 years? ‘It is important to keep your good spirit, despite ups and downs, periods of stagnation and progress; the time for recovery may seem endless but you must believe in it and avoid letting yourself be discouraged.’

Five years later, she admits to no longer think about it. ‘Today everything is going well on a day-to-day basis and I do not think of my hand anymore. I do not feel like I've had a stroke.’

## Data Availability

The original contributions presented in the study are included in the article/Supplementary Material, further inquiries can be directed to the corresponding author.
