# Supplementary material for: Exceptional improvement in chronic stroke through Guided Self-rehabilitation Contract: a case report study
Source: Front Rehabil Sci. 2024 Sep 18;5:1385483. doi: 10.3389/fresc.2024.1385483 (PMC11447270; doi:10.3389/fresc.2024.1385483)
Supplement: Supplementary file 3 [file Datasheet1.pdf]

Supplemental Appendix: Explanation of videos and MFS

Explanations of videos 1 and 2 and guidelines for the Modified Frenchay Scale (MFS) (38)

1- Video 1 and video 2: Both videos display the performance on the Modified Frenchay Scale (MFS), which consists of video-taping ten activities of daily living (4 uni-manual activities using the paretic hand and 6 bimanual activities, in which the paretic hand assists the other hand) and rating each of them on a ten-point visual analog scale based on video-review. In that visual analog scale, zero means no movement, 10 is perfect achievement of the task and 5 a task barely accomplished.

Video 1 shows **the first 30 seconds** of the patient performance on February 2015, 16 months post stroke; video 2 shows the performance in December 2018, after 4 years of Guided Self-rehabilitation Contract.

2- Guidelines for the Modified Frenchay Scale: MFS
Required equipment:

- Jam jar of about 8 cm in diameter
- Big bottle of about 8 cm in diameter, filled up to one-third
- Small bottle of about 6 cm diameter, filled up to one-third
- Empty solid cup about 7 cm diameter
- Notepad or slate
- 20 cm ruler and pen
- Three clothes pins
- Comb
- Tube of toothpaste and toothbrush
- Knife and fork
- Broom

Guidelines to raters: Place the objects in front of the patient in the order of tasks, from left to right in an arc shape, approximately one arm length away from the patient.

- Ask the patient to pull up the sleeve on the paretic upper limb.

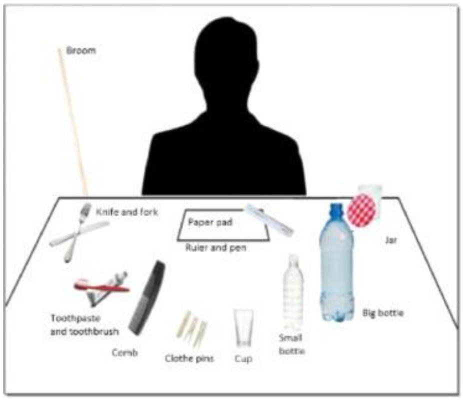

- Perform the tasks 1 by 1, always in the same order.
- Unimanual tasks should be performed with the paretic hand only, *without* help from the other hand.
- If the patient cannot reach the object, try again after placing the object next to him close to the edge of the table (task rating will necessarily be <5).
- Overactive muscles of the paretic hand may be self-stretched before each task.
- The patient should *not* stand up to perform the task.

Suggestions for rating: *Half points are allowed*

- 0: No movement
- 1: Movement initiated with ≥1 movement component
- 2: Task still closer to not being achieved than to being achieved
- 2.5: Task midway between being achieved and not
- 3: Four movement components still incomplete (e.g., for a unimanual task: shoulder flexion, elbow extension, elbow supination, and finger extension, which deducts 4 half points from 5)
- 4: Task close to being achieved but 2 movements components still incomplete (two half points deducted from 5)
- 5: Task barely accomplished
- 6: *Sense of security*: no doubt that the task was going to be achieved but task completed with difficulty and slowness
- 7: *Sense of smoothness*: task accomplished with some smoothness but still slow
- 8: *Sense of speed*: task completed fast but not normal
- 9: Almost normal
- 10: Normal performance

MFS Tasks and Scoring

1. Open and close the jam jar using both hands (paretic hand holds the jar)
Specific comment: Hold the jar above the table (not in contact with the table), otherwise rate <5

.**2. Reach, pick up and release the large bottle using paretic hand only**
Specific comment: Grasp the bottle by the side from its initial position at arm length; if bottle is grasped from the tip, or from table edge only, rate <5.

3. Reach, pick up, and release the small bottle using paretic hand only
Specific comment: Grasp the bottle by the side from its initial position at arm length; if bottle is grasped from the tip or from table edge only, rate <5.

4. Reach, pick up the cup, and bring to mouth using paretic hand only
Specific comment: Grasp the cup by the side from its initial position at arm length; all components of the movement must be barely completed to achieve 5, including external rotation of the shoulder to bring cup to mouth.

5. *Rule a line with the ruler using both hands (the paretic hand holds the ruler)*
Specific comment: If ruler is stabilized by paretic hand closed, rate 5. If ruler is barely stabilized by paretic hand almost open, rate 6.

6. Clip 3 clothespins to the notepad using both hands (paretic hand holds and clips pins)
Specific comment: If paretic hand clips only 1 out of the 3 clothespins, rate 5

.**7. Reach, pick up the comb, and mimic combing using the paretic hand only**
Specific comment: Pick up comb from initial position at arm length, if comb is picked up from table edge only, rate <5

.**8. Put toothpaste on toothbrush using both hands (paretic hand holds and presses on tube)**

9. Pick up knife and fork using both hands and mimic cutting (paretic hand holds the fork)
Specific comment: To reach 5, the paretic hand must both grip *the fork firmly enough (with Digits 3, 4, 5 and thumb), point the index finger along the fork axis, and have sufficient elbow pronation and shoulder abduction/internal rotation to be able to mimic planting fork.*

10. Sweep the floor with the broom using both hands

Total score____
