# Supplementary material for: Exceptional improvement in chronic stroke through Guided Self-rehabilitation Contract: a case report study
Source: Front Rehabil Sci. 2024 Sep 18;5:1385483. doi: 10.3389/fresc.2024.1385483 (PMC11447270; doi:10.3389/fresc.2024.1385483)
Supplement: Supplementary file 4 [file Datasheet2.pdf]

APPENDIX – ASSESSMENT OF THE FINGER FLEXORS

Position of the Patient

Patient is seated, relaxed and resting against the back rest, in a chair without arms. Opposite arm is in a comfortable position. Examiner sits beside the patient.

1. Starting finger position: from ‘zero, theoretical position of minimal stretch of the muscle’ (as in the initial description of the Tardieu Scale (29), i.e. fingers fully flexed, second phalanx of DIII parallel to the metacarpal bone; see figure below.
2. Shoulder in neutral position (i.e. arm hanging vertically)
3. Elbow flexed at 90°
4. Wrist and metacarpal joints *in neutral position* (i.e. 0° in terms of anatomical angles), maintained by the first hand of the examiner
5. The second hand grabs the fingers trying to have the examiner’s fingers facing the palmar aspect of the subject fingers
6. Perform finger extension:
   I. Slowly and strongly- until movement arrest - for $\mathbf{X_{V1}}$ of Tardieu (maximal passive clinical extensibility of muscle)
   II. As fast as possible for $\mathbf{X_{V3}}$ and **Y** of Tardieu (X_{V3}, angle of catch, reflecting the threshold of the stretch reflex)
   iii. Have the patient perform the best possible finger extension for measurement of $\mathbf{X_A}$, the Active Range of Motion angle against the resistance from the finger flexors

The fulcrum (rotation axis) *is* the metacarpophalangeal joint of the third finger. As a reminder, goniometers are only used for the active parts of the Five Step Assessment. The passive parts (Tardieu Scale itself for the measurements of XV1 and XV3) are performed using visual assessments only, without goniometry.

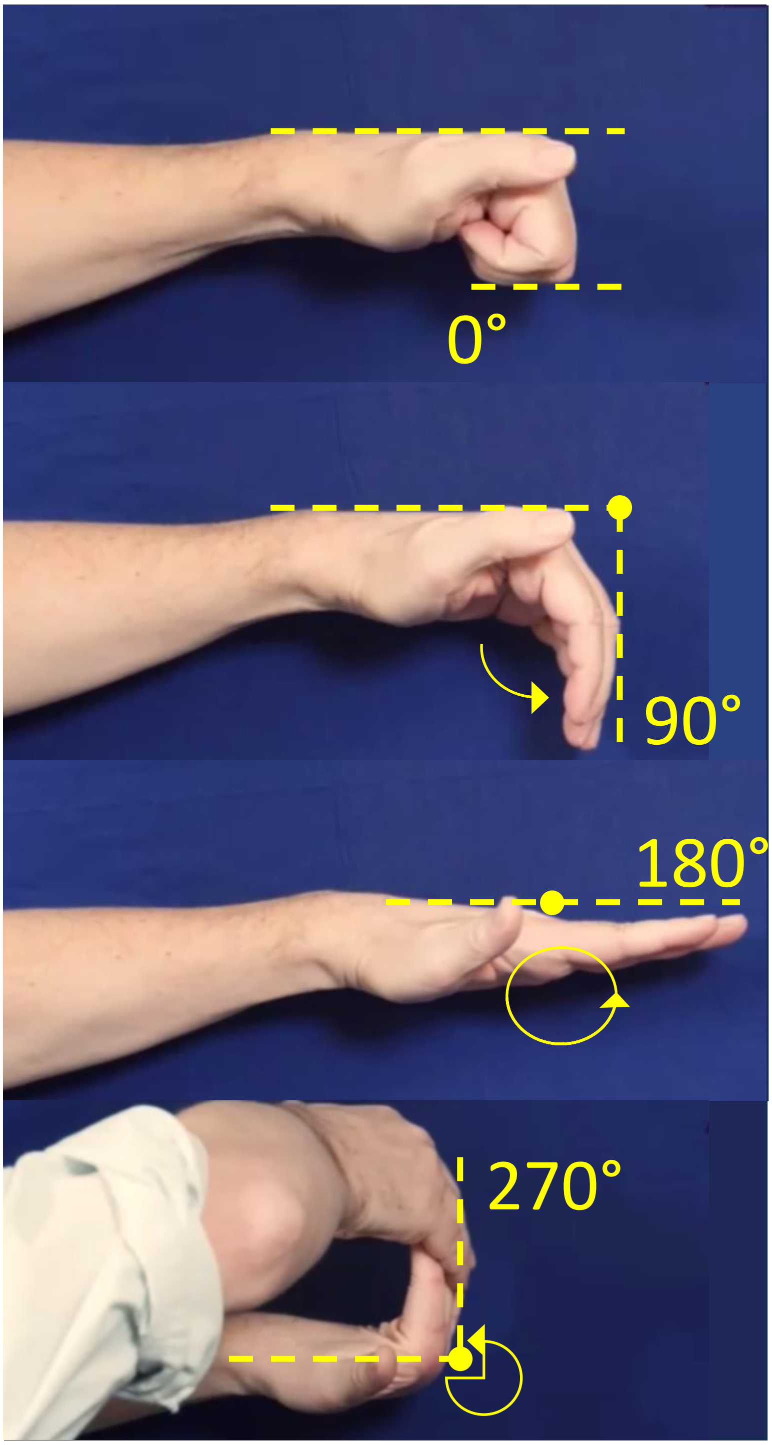

Figure - Measurement of stretch angles for the finger flexors
