# Supplementary material for: Exceptional improvement in chronic stroke through Guided Self-rehabilitation Contract: a case report study
Source: Front Rehabil Sci. 2024 Sep 18;5:1385483. doi: 10.3389/fresc.2024.1385483 (PMC11447270; doi:10.3389/fresc.2024.1385483)
Supplement: Supplementary file 5 [file Table1.docx]

Supplemental Table 1

**Table 1**: Passive maximal amplitudes X_V1_ at onset (i) and end (f) of GSC (X_V1-i_, X_V1-f_) for six antagonists and angles of match XA at onset (i) and end (f) of GSC, X_A_ (X_A_-i, X_A_-f) against these antagonists; (*) Value considered within the margin of measurement error

*Long Head Triceps*

180°

130°

180°

**+50°**

*Shoulder flexion (elbow flexed)*

90°

180°

**+90°**

*Sub-scapularis*

180°

160°

180°

**+20°**

*Shoulder external rotation*

100°

180°

**+80°**

*Elbow flexors*

180°

179°

180°

*Elbow extension*

140°

180°

**+40°**

*Pronator teres*

180°

176°

180°

*Supination (elbow extended)*

90°

180°

**+90°**

*Wrist flexors*

180°

160°

180°

**+20°**

*Wrist extension*

110°

180°

**+70°**

*Finger flexors*

270°

250°

270°

**+20°**

*Hand opening*

0°

270°

**+270°**

**Initial active**

**amplitude (X**

**A-i**

**)**

**Final active**

**amplitude (X**

**A-f**

**)**

**ΔX**

**A**

**Targeted**

**antagonist muscle**

**Physiologic**

**Amplitude (X**

**N**

**)**

**Initial passive**

**amplitude (X**

**V1-i**

**)**

**Final passive**

**amplitude (X**

**V1-f**

**)**

**ΔX**

**V1**

**Active movement**

**against the antagonist**

**+1°** (*)

**+4°** (*)
